# Neuroprotective Role of the Nrf2 Pathway in Subarachnoid Haemorrhage and Its Therapeutic Potential

**DOI:** 10.1155/2019/6218239

**Published:** 2019-05-02

**Authors:** Ardalan Zolnourian, Ian Galea, Diederik Bulters

**Affiliations:** ^1^Department of Neurosurgery, Wessex Neurological Centre, University Hospital Southampton, Tremona Road, Southampton SO16 6YD, UK; ^2^Clinical Neurosciences, Clinical & Experimental Sciences, Faculty of Medicine, University of Southampton, Tremona Road, Southampton SO16 6YD, UK

## Abstract

The mechanisms underlying poor outcome following subarachnoid haemorrhage (SAH) are complex and multifactorial. They include early brain injury, spreading depolarisation, inflammation, oxidative stress, macroscopic cerebral vasospasm, and microcirculatory disturbances. Nrf2 is a global promoter of the antioxidant and anti-inflammatory response and has potential protective effects against all of these mechanisms. It has been shown to be upregulated after SAH, and Nrf2 knockout animals have poorer functional and behavioural outcomes after SAH. There are many agents known to activate the Nrf2 pathway. Of these, the actions of sulforaphane, curcumin, astaxanthin, lycopene, *tert*-butylhydroquinone, dimethyl fumarate, melatonin, and erythropoietin have been studied in SAH models. This review details the different mechanisms of injury after SAH including the contribution of haemoglobin (Hb) and its breakdown products. It then summarises the evidence that the Nrf2 pathway is active and protective after SAH and finally examines the evidence supporting Nrf2 upregulation as a therapy after SAH.

## 1. Introduction

SAH is a devastating condition and is associated with high levels of morbidity and mortality [1]. Despite advances in treatment, 40% of SAH survivors remain dependant due to physical disability, behavioural, and cognitive disturbances [2–4] and even amongst those who are seemingly independent, 50% suffer from neurocognitive deficits [5].

The mechanisms leading to poor outcome after SAH are complex and multifactorial. They include early brain injury [6] and a host of different ensuing processes including oxidative stress [7], inflammation [8], spreading depolarisation [9], and microscopic [10] and macroscopic vasospasm [11].

The purpose of this review is to evaluate the evidence for pharmacological augmentation of the Nrf2 pathway as a treatment for patients with SAH. In order to do this, we have first reviewed the mechanisms underlying poor outcomes after SAH, then reviewed the Nrf2 pathway, before considering how the Nrf2 pathway applies to SAH, and finally reviewed the evidence for specific compounds known to upregulate Nrf2 activity.

## 2. Mechanisms of Injury after SAH

The mechanisms leading to poor outcome after SAH can be categorised into primary and secondary injury in a way analogous to head injury, where the immediate damage that occurs directly from the insult is classed as primary and any further subsequent indirect damage as a result of the processes initiated by the insult is secondary. The important clinical distinction is the presumption that secondary injury is potentially treatable, whereas primary injury is not. While this concept is well established in head injury, the terms are not as widespread in SAH where the immediate injury is often classed together with subsequent events in the first 72 hours as early brain injury, and all ensuing events are considered separately. The latter events could also be grouped together as delayed brain injury. While some mechanisms like cerebral vasospasm clearly follow this classification, there are limitations, and others such as spreading depolarisation straddle both time periods.

### 2.1. Early Brain Injury

At the time of SAH, intracranial pressure rises to that of diastolic arterial pressure or higher [12, 13]. This will in turn result in reduction of cerebral blood flow and cerebral perfusion pressure [14–16]. Consequently, cerebral autoregulation is disturbed [17, 18]. Blood-brain barrier (BBB) dysfunction [19], cerebral oedema [20, 21], and neuronal cell death [22] all take place within 72 hours from injury. Altered ionic homeostasis, excitotoxicity, thrombin activation [23], vascular integrity degradation [24], oxidative stress [7], inflammation [8], elevated matrix metalloproteinase-9 (MMP9) [25], and activation of the nitric oxide synthase (NOS) pathway [26, 27] are all seen.

### 2.2. Cerebral Vasospasm

Following lysis of red blood cells in the subarachnoid space, the central nervous system is exposed to high levels of Hb and its degradation products which lead to narrowing of the cerebral vessels and development of delayed cerebral ischaemia (DCI) in 30% of patients [11, 28]. In addition, DCI is diagnosed clinically in patients with reduced consciousness state and/or neurological deficits, the exact cause of which remains unclear. It is, however, part of the secondary brain injury and is most likely dependent on the initial pathological process after SAH.

Following SAH, oxyhaemoglobin (OxyHb) has been shown to induce vasoconstriction in animal models [29–31]. OxyHb is thought to cause arterial contraction directly and via the production of reactive oxygen species (ROS), since both Hb and ROS scavenge free nitric oxide [32]. OxyHb also decreases the activation of K^+^ channels which leads to an intracellular surge of calcium and promotes vasoconstriction [32, 33]. However, the exact pathophysiology remains unknown. The underlying mechanisms are thought to be multiple [34] and include oxidative stress [35], neuronal apoptosis [36, 37], decreased production of nitric oxide [6, 26], increased endothelin-1 [38, 39], calcium [40, 41], prostaglandin [42], thromboxane levels [32, 43], and spreading cortical depolarisation [9, 44]. This ultimately results in cerebral ischaemia peaking between days 4 and 14 postictus [11, 45]. Regardless of the predominating mechanism, there is a clear relationship between cerebral vasospasm and the amount of subarachnoid blood [46, 47].

However, macrovascular vasospasm does not always correlate directly with the development of DCI. In fact, transcranial Doppler and angiographic studies have only shown a positive predictive value of 57% and 76%, respectively [48]. When combined together, this is as high as 67% [49]. The degree to which macroscopic vasospasm influences outcome was further put in doubt by studies of the endothelin receptor antagonist, clazosentan, which demonstrated large consistent reductions in angiographic vasospasm without associated improvement in clinical outcomes [50].

It has therefore been proposed that poor outcome is conferred by spasm of the microvasculature rather than macroscopic vasospasm seen on angiography [51]. Spasm of arterioles has been shown in animal models of SAH [52–54]. In a mouse SAH model, vasoconstriction of arterioles was seen in more than 70% of subjects starting at three hours and persisting for at least three days after haemorrhage. An inverse correlation between the size of the arterioles and the extent of vasoconstriction was observed. In addition, 30% of the arterioles were occluded by microthrombi. The vessels were more likely to be severely constricted if there was already evidence of microthrombi. These findings may explain the poor cerebral perfusion pressure which may lead to DCI after SAH [55]. In a dog SAH model, morphometric examination of the internal diameter of arterioles revealed significant reduction, with marked increase in vessel wall thickness three and seven days after SAH [53].

An intraoperative study used orthogonal polarisation spectral imaging during aneurysm surgery to visualise the response of the small cortical vessels to hypercapnia. Patients with visible blood clot who underwent early surgery had a more pronounced vasoconstrictive response (39%) compared to the group without visible blood clot who had late surgery (17%) and patients with unruptured aneurysms (7%) [10]. In addition, microvascular vasospasm in patients with DCI has been demonstrated by measuring the cerebral circulation time from digital subtraction angiograms. Prolonged cerebral circulation time as a marker of microvascular vasospasm was shown to be directly related to decreased regional cerebral blood flow [56].

These findings are consistent with a postmortem study of 53 aneurysmal SAH patients which demonstrated extensive cortical and hypothalamic infarctions with histologic evidence of microangiopathy [57]. Small cortical and hypothalamic infarcts have been noted in other postmortem studies [56]. The relationship between microvascular vasospasm and DCI may also explain why up to 25% of patients have CT evidence of infarction in a different location to the spastic artery or have no evidence of macrovascular spasm [58, 59].

### 2.3. Oxidative Stress

After SAH, free extracellular Hb undergoes oxidation to methaemoglobin (MetHb), which then degrades into haem. Free haem is toxic and acts as a catalyst for formation of ROS causing oxidative stress. Haem toxicity is exerted by its proinflammatory properties as well as damage caused by ROS leading to modification of lipids, carbohydrates, and nucleotides with eventual cell death affecting both neurons [60] and endothelial cells [61].

Following conversion of OxyHb to MetHb, superoxide radicals are released which convert to hydroxyl radicals [31]. Human studies show evidence of an increase in oxidative stress and lipid peroxidation in both CSF and serum as early as three days after SAH [7, 62]. These increases are more commonly seen in patients with poor outcome [63]. Blocking lipid peroxidation with a non-glucocorticoid aminosteroid, tirilazad, has been assessed in five randomised clinical trials. However, meta-analysis of these trials showed no improvement in clinical outcomes in patients [64].

ROS produce vasoactive lipids via reactions with arachidonic acid, resulting in vasoconstriction. Furthermore, free radical oxidation of bilirubin and biliverdin leads to formation of bilirubin oxidation products [65]. The accumulation of these products and bilirubin in the CSF has been shown to be associated with DCI and vasospasm after SAH [66].

Oxidative stress has been linked to the activation of protein kinase C [6, 67] and Rho kinase [6], both of which are involved in smooth muscle contraction. Protein kinase C plays an important role in vascular smooth muscle cell growth as well as vascular remodelling, as seen in vasospasm [32]. Intra-arterial fasudil (a Rho kinase inhibitor) has been successful in experimental SAH [68], and it has also been used in patients reducing average arterial circulation time with potential reduction in cerebral vasospasm [69]. However, the clinical benefit of this remains uncertain.

### 2.4. Inflammation

Following SAH, free Hb released in the subarachnoid space stimulates rapid expression of cell adhesion molecules by endothelial cells, attracting neutrophils [1]. These cells are subsequently trapped in the subarachnoid space and may be implicated in vasospasm through the enzymatic activity associated with the oxidative burst. Constant inflammatory stimulation may result in chronic inflammation involving lymphocytes and monocytes [70]. Monocytes invade the injured tissue to become macrophages. Lymphocytes and macrophages release inflammatory cytokines including IL-1*β*, IL-6, and TNF-*α* in the CSF [71]. Blood-brain barrier breakdown further accentuates release of inflammatory cytokines, which peaks at day seven postictus. Increasing levels of IL-1, IL-6 and TNF-*α* have been shown to be associated with poor outcome [72–75], and blockade with interleukin-1 receptor antagonist (IL-1RA) has been shown to reduce this [76–78]. Inflammation in the brain is also linked to post-SAH systemic inflammatory response syndrome and organ failure [1]. Neuroinflammation has been shown to be associated with cognitive dysfunction in other disorders [79, 80] so this could be a putative mechanism underlying such deficits in SAH patients.

### 2.5. Cortical Spreading Depolarisation

Cortical spreading depolarisation (CSD) refers to slow waves of near-total neural depolarisation with resultant cellular swelling due to the influx of cations across the cell membrane. This exceeds the ATP-dependent Na^+^ and Ca^2+^ pump activity which leads to shrinkage of the extracellular space due to water influx [9]. CSD can be induced by a variety of means [81]. It can occur immediately after SAH and even up to two weeks from the cerebral insult [82]. After SAH, it is usually triggered by high K^+^ [83, 84] released from degraded erythrocytes or alternatively from cortical injury from the initial bleed.

The normal response to a short episode of CSD is hyperaemia. However, in SAH following a single wave of CSD associated with OxyHb [85, 86], reduced nitric oxide concentration [83, 87], or endothelin-1 [88], the normal response is reversed [89]. Moreover, CSD triggers vasoconstriction resulting in cortical spreading ischaemia (CSI) [85, 90] which may lead to cortical necrosis [86]. In addition, prolonged or repetitive CSD can lead to tissue damage without CSI, simply by the increased metabolic demand, i.e., increased oxygen utilisation [91].

As well as in animal studies [84–86, 92, 93], CSD and CSI have been demonstrated in SAH patients [9, 44, 94]. The multicentre Co-Operative Studies on Brain Injury Depolarizations (COSBID) showed a strong association between CSD and DCI for the first time in humans. 13/18 patients (72%) demonstrated signs of CSD on electrocorticography recorded via a subdural strip over the cerebral cortex that was placed during the craniotomy and was monitored for ten days. Seven patients developed DCI. CSD had positive and negative predictive values of 86% and 100%, respectively. This study also revealed that DCI may occur in the absence of radiological vasospasm but is still associated with clusters of spreading depolarisation, meaning that large vessel spasm is not the main driver of DCI [44]. Moreover, spreading depolarisation upregulates multiple genes, such as haem-oxygenase-1 (HO-1) [95], which could be protective and might explain why in some patients subsequent ischaemia does not take place.

## 3. Nuclear Factor-Erythroid 2- (NF-E2-) Related Factor 2 (Nrf2)

Nuclear factor-erythroid 2- (NF-E2-) related factor 2 (Nrf2) is a redox-sensitive transcription factor belonging to the cap'n'collar (CNC) subclass of the basic leucine zipper region containing the protein family. It binds to a specific DNA site, the antioxidant response element (ARE), regulating transcription of an array of detoxifying or antioxidant enzymes. These include gamma-glutamylcysteine synthetase, superoxide dismutase, catalase, glutathione reductase, thioredoxin reductase, peroxiredoxins, and glutathione S-transferase (GST-*α*1) [96, 97]. It also regulates degradation of red blood cells, Hb, haem, and iron through transcriptional upregulation of CD36 [98], haptoglobin (Hp) [99], haemopexin [100], HO-1 [101], and ferritin [102].

Hp has received particular attention following SAH. It is the fourth most abundant plasma protein and is synthesised in the liver and reticuloendothelial system [103]. Outside the brain, extracellular Hb is immediately bound to Hp via an extremely strong interaction [104]. Hp is not synthesised in the brain under normal physiological conditions and diffuses into the CNS. In the pathologic state, it is expressed by astrocytes [105] and oligodendrocytes [99]. The Hb-Hp complex is recognised by the CD163 membrane receptor, leading to internalisation [106]. In the CSF, concentration of Hp is two orders of magnitude lower than in the circulation, which is insufficient to bind all Hb arising from the clot [107]. The CD163 uptake system is saturated, as evidenced by the presence of the Hb-Hp complex in the CSF after SAH [107].

Although no suitably powered human study of CSF Hp levels has been done to assess its relationship with outcome, the Hp phenotype has been shown to be important in determining outcome after SAH. Human Hp is composed of two peptide chains: *α* and *β*. There are two different *α* alleles giving rise to three different phenotypes: *α*1*α*1, *α*1*α*2, and *α*2*α*2 [108]. The Hp *α*2 genotype has been shown to be associated with cerebral vasospasm [109–112], cerebral salt wasting [113], and poor outcome [113, 114]. In addition to the Hp-dependent pathway, there are other less efficient scavenging systems such as cubilin and megalin [115] and downstream haem clearance via the haemopexin-CD91 pathway [116].

### 3.1. Nrf2 Regulation

**Figure 1:**
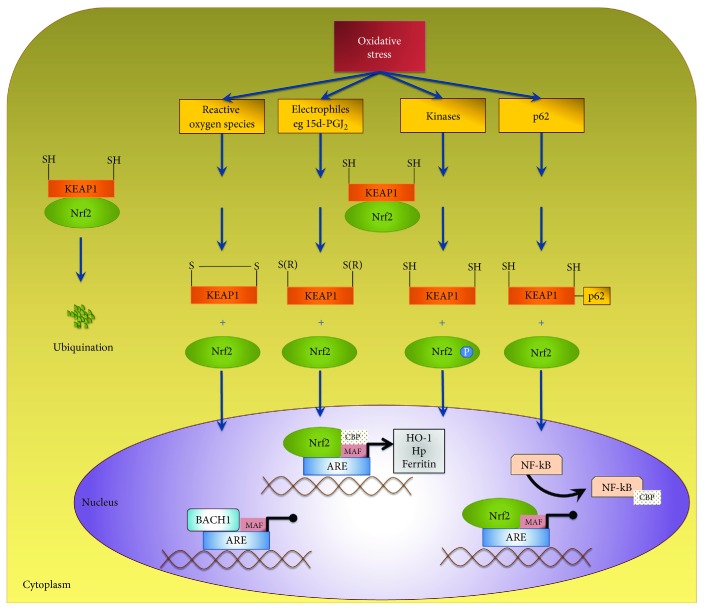
Nrf2 regulation. Nrf2 is a redox-sensitive transcription factor that is bound to KEAP1 under physiological condition. KEAP1 is an intracellular redox sensor and targets Nrf2 for ubiquitination. Following oxidative stress, four different mechanisms result in dissociation of KEAP1 from Nrf2. These four mechanisms are as displayed in order: (1) oxidation of cysteine residues by lower molecular weight reactive oxygen species, (2) covalent modification of cysteine residues by electrophiles such as NF-*κ*B-induced cyclopentenone prostaglandins, (3) phosphorylation of Nrf2 at Ser40 by protein kinase C and PERK, and (4) protein-protein interaction between p62 and KEAP1. Free of KEAP1, Nrf2 translocates into the nucleus where it binds to antioxidant response elements in DNA to mediate transcription of key proteins. Nrf2 requires the binding partners MAF and CBP to initiate transcription. BACH1 competes for MAF and NF-*κ*B competes for CBP. Overall, the equilibrium between the two transcriptions factors BACH1 and Nrf2 determines overall transcription of the downstream genes.

During normal physiological conditions, Nrf2 is bound to the Kelch-like ECH-associated protein 1 (KEAP1) in the cytoplasm [96] (Figure 1). KEAP1 is a homodimer with three major domains, one of which facilitates ubiquitination of Nrf2 [117]. KEAP1 is an intracellular redox sensor. In response to oxidative stress as happens after SAH, key cysteine residues on KEAP1 are oxidised [117]; in addition, Nrf2 is phosphorylated on Ser40 by protein kinases [118–120]. One or both events lead to Nrf2 release from KEAP1; Nrf2 then translocates into the nucleus, to act as a transcription factor [121] (Figure 1). Furthermore, p62 is a protein that has six domains, one of which is the KEAP1-interacting region (KIR) [122–124]. The protein p62 has been identified to be involved in activation of Nrf2 [124, 125] by inhibiting KEAP1-mediated Nrf2 ubiquitination, leading to stabilisation and a rise in Nrf2 levels [123, 124].

While KEAP1, as an intracellular redox sensor, regulates the transcriptional response to oxidative stress through Nrf2, this is balanced by the activity of other transcription factors such as nuclear factor kappa-light-chain-enhancer of activated B cells (NF-*κ*B) and BTB (broad complex, tramtrack, bric-a-brac) and CNC homology 1 (BACH1). NF-*κ*B and Nrf2 both need to bind CREB-binding protein (CBP) to exert their transcriptional effects, so activation of NF-*κ*B can inhibit the Nrf2 transcriptional programme by limiting the availability of CBP [126] (Figure 1). BACH1 competes with Nrf2 to bind to ARE and can repress the effects of Nrf2 [127]. It is the equilibrium between Nrf2 and BACH1 that determines the production of genes under their control (Figure 1).

### 3.2. Nrf2 in the Brain

Nrf2 is expressed in the central nervous system (CNS), and it is upregulated in response to inflammation and cerebral insults [128]. Nrf2 therefore plays a key role in conditions where inflammation is the hallmark. For example, both ischaemic and haemorrhagic strokes share some common pathophysiologic pathways with SAH. Outcomes after both have been shown to be ameliorated by activation of the Nrf2 pathway.

A number of studies of ischaemic stroke models [129–131] have shown that Nrf2 levels rise soon after the onset of stroke. In a permanent ischaemic stroke model, this was as early as three hours from the ictus and peaked at 24 hours postinsult [132]. In a transient stroke model, a significant increase in Nrf2 was seen at two hours with a peak at eight hours postreperfusion and decreasing at 24 hours. Nrf2 levels were measured in both the peri-infarct and the core ischaemic regions with a marked increase in the peri-infarct area [129]. Increased levels of peri-infarct Nrf2 is most likely due to the increased oxidative stress in this region [129]. Furthermore, Nrf2 expression has been demonstrated in neurons, astrocytes, leukocytes, and microglia [129, 130, 132]. Nrf2 knockout models are also associated with poorer neurological recovery [133, 134].

The Nrf2 pathway is also activated following intracerebral haemorrhage (ICH) in mice. HO-1 was shown to be upregulated after 24 hours, peaking at five days with a return to baseline on day eight [101]. Nrf2 knockout mice suffered more severe neurological deficits. There was larger injury volume, increase in leukocyte infiltration, production of ROS, DNA damage, and cytochrome c release during the critical early phase of the post-ICH period with poorer neurological recovery [135].

Nrf2 is also protective against hemin toxicity. Rat astrocyte cultures were pretreated with Hb or vehicle, and then exposed to hemin, the degradation product of Hb. Pretreated astrocytes showed resistance to toxicity induced by hemin. Pretreatment with Hb was shown to induce Nrf2 and HO-1, and the latter led to haem catabolism, in keeping with the protective effect of Hb pretreatment. In support of this mechanism, the protective effect of Hb pretreatment was lost in Nrf2 knockdown cells [136].

### 3.3. Nrf2 in SAH

Experimental data have shown that Nrf2 expression is upregulated in the basilar artery of rats after SAH [137]. This was observed in the nucleus and cytoplasm of endothelial cells, smooth muscle cells, and adventitial cells on day five following SAH, demonstrating marked activation of the Nrf2 system [137].

Chen and colleagues using a rat SAH model demonstrated that Nrf2 expression is also increased in the cortex at 12 hours, 24 hours, and 48 hours postinjection of blood compared to controls, with a peak at 24 hours postinjection [138].

Deletion of Nrf2 has been shown to be associated *in vitro* with an increased inflammatory response in cultured murine astrocytes. In a model utilising primary cultured astrocytes exposed to OxyHb, downstream inflammatory cytokines such as TNF-*α*, IL-1*β*, and IL-6 as well as MMP9 were significantly higher in Nrf2 knockout mice, and this was accompanied by NF-*κ*B upregulation [139]. The effect of Nrf2 knockout was also studied in an *in vivo* mouse SAH model. Brain oedema and neural cell death after SAH were measured and compared between wild-type mice and Nrf2 knockout mice. Nrf2 deficiency increased brain oedema and neural cell death at 24 hours after SAH. Neurological deficits as measured by posture, grooming, and ambulation were also markedly worse in Nrf2-deficient mice [140].

The pathophysiology of SAH involves oxidative stress and inflammation. The redox state can be assessed using malondialdehyde (MDA) levels and the GSH/GSSG ratio. MDA is a lipid peroxidation product and is elevated after oxidative stress [141]. The GSH/GSSG ratio is thought to represent antioxidative capacity and is decreased in many inflammatory CNS disorders [142, 143]. Nrf2 knockout mice were found to have higher MDA levels and a lower GSH/GSSG ratio. Furthermore, inflammatory cytokines including TNF-*α* and IL-1*β* were significantly increased [140]. Cerebral vasospasm at 24 hours after experimental SAH in Nrf2 knockout mice was not significantly different from wild-type animals. This could suggest that cerebral vasospasm may occur independently of inflammation and oxidative stress. However, another study showed that Nrf2 upregulation was associated with a decreased rate of vasospasm in a rat model of SAH [144]. It is possible that the different results in vasospasm observed between these two studies are due to compensatory mechanisms in the Nrf2 knockout mice, or other technical differences.

## 4. Therapeutic Potential of Nrf2 Activators in SAH

There are a large number of known activators of the Nrf2 system. All act by binding KEAP1 releasing Nrf2, which translocates to the nucleus leading to increased transcription. Nrf2 activators are broadly classified as electrophilic cysteine-reactive compounds and nonelectrophilic Keap1-Nrf2 protein-protein interaction inhibitors. Most well-established compounds fall into the former category. However, many are pleiotropic and their primary mechanism of action remains controversial. There are also efforts being made to develop new more selective compounds of the latter category. These have the potential to be more potent inducers with less cross-activation of other pathways [145].

SAH represents an ideal condition for these treatments. With the wide range of proteins upregulated by Nrf2, it can be postulated that Nrf2 activation may have beneficial effects on any of the described secondary mechanisms underlying poor outcome. The magnitude of this effect is likely to be dictated by the timing of administration of the Nrf2 activator.

Following SAH, DCI occurs no earlier than three days after the event and is not seen beyond 21 days. Even if the mechanisms leading to it are initiated earlier, Nrf2 preconditioning has been shown to be protective in ischaemic stroke. Therefore, a case can be made for administration as late as 72 hours after SAH, which would be consistent with most previous drug studies in SAH [146].

Prevention may require earlier treatment. Most discussed mechanisms are either initiated or worsened by Hb. Given that it takes days for red cell lysis, and intracellular Hb to be released, CSF Hb levels progressively rise from day one to day six after SAH [147]. This also offers a therapeutic window during which the Nrf2 system can be preemptively fully induced to ameliorate this. This is likely to have effects on macro- and microvascular spasm and Hb-mediated components of oxidative stress and inflammation.

However, other aspects of inflammation and oxidative stress may result directly from early brain injury. These and indeed early brain injury itself would require earlier treatment still. Preconditioning would offer the greatest chance of benefit but is clearly not practical.

Therefore, aiming for treatment in patients at the earliest available opportunity, but accepting treatment up to 72 hours after SAH where patients do not present immediately, would seem a pragmatic approach, although early phase studies may benefit from shorter recruitment windows to increase the chance of observing an effect. Here, we have reviewed all agents that have been tested and have shown therapeutic potential in SAH. We have summarised the characteristics of each Nrf2 activators in Table 1 and listed the individual animal studies of Nrf2 activators in experimental SAH in Table 2.

## 5. Sulforaphane

Sulforaphane (SFN), 1-isothiocyanate-(4R)-(methylsulfinyl) butane, is a widely studied isothiocyanate. SFN stabilizes Nrf2 by inhibiting its ubiquitination. Oxidation of critical cysteine residues of KEAP1 by SFN appears to be essential [117], but subsequent mechanistic steps are a matter of controversy. While it has been widely believed that Nrf2 stabilisation occurs by freeing Nrf2 from KEAP1, as happens naturally when KEAP1 cysteines are oxidised, this has been recently challenged with a suggestion that Nrf2 is stabilised in complex with KEAP1 in the nucleus [148]. Although Nrf2 phosphorylation at Ser40 via protein kinase pathways may be involved in its stabilisation during oxidative stress [118–120], this is not implicated in chemically induced stabilisation of Nrf2 by SFN [148]. In line with stabilisation of Nrf2, SFN leads to upregulation of Hp expression in the periphery [149] and in the brain [99].

The effect of SFN has been assessed in an *in vitro* SAH model. Rat aortic arch cells were exposed to OxyHb and SFN for 48 hours. Levels of Nrf2 and Nrf2-regulated genes including HO-1 and NQO1 (NAD(P)H:quinone oxidoreductase 1) were significantly increased and further upregulated when exposed to SFN. In addition, the concentrations of inflammatory cytokines IL-1*β*, IL-6, and TNF-*α* were markedly reduced in the SFN group [150].

The effects of SFN after SAH *in vivo* were first assessed by Chen et al. [138]. Autologous blood was injected in the prechiasmatic cistern of rats. Intraperitoneal SFN was injected at 30 minutes, 12 hours, and 36 hours. mRNA expressions of HO-1, NQO1, and GST-*α*1 were measured in the cortex of the animals after 48 hours. SAH led to increased expression of HO-1, NQO1, and GST-*α*1 in the rat cortex. A further significant increase was seen after treatment with SFN demonstrating that SFN has the capacity to increase Nrf2 activity even in an already highly induced state. Brain oedema, BBB permeability, and apoptotic cell death were all reduced following treatment with SFN. Treatment with SFN was associated with a reduction in motor deficits in the rotarod test performed at 24 hours. These results demonstrate that SFN upregulates the Nrf2-ARE pathway after SAH and reduces early brain injury. It was associated with improved early function, although further study would be needed to demonstrate if this translates to better long-term outcomes [138].

More recently, an experimental study [144] assessed the effects of SFN on cerebral vasospasm after SAH. Autologous blood was injected in the cisterna magna of rats and repeated after 48 hours. Intraperitoneal injection of SFN was then performed every 24 hours from 30 minutes after induction of SAH to the third and last day of the experiment. Tissues were harvested three days after SAH. Cross-sectional areas of basilar arteries showed a significant difference between the SFN and untreated SAH groups. SFN administration increased the mRNA expression levels of Nrf2, HO-1, and NQO1 as well as significantly upregulating Nrf2 in endothelial and smooth muscle cells. The inflammatory cytokines IL-1*β*, IL-6, and TNF-*α* were significantly reduced following treatment with SFN. SFN was also found to ameliorate the behavioural deficits of rats following SAH as demonstrated by improvement in appetite and activity scores, but this was only performed once at three days following SAH. Overall, these results confirm that early administration of SFN upregulates the Nrf2 pathway after SAH, reduces vasospasm, and improves function at least early after SAH [144].

In addition to reducing oxidative stress in the subarachnoid space and consequently reducing cerebral vasospasm, SFN may have a beneficial effect on the ischaemia which can follow cerebral vasospasm. In a rat middle cerebral artery (MCA) occlusion, stroke model preconditioning with SFN one hour prior to stroke and reperfusion after four, 24, and 72 hours upregulated Nrf2 and HO-1 expression leading to attenuation of BBB disruption, lesion progression as assessed by magnetic resonance imaging between 24 and 72 hours, and neurological dysfunction (based on motility, grasping reflex, and placing reaction) [151]. In a rat ischaemic stroke model, SFN was shown to reduce infarct volume following temporary occlusion of the left common carotid artery or MCA. Animals in the treatment group were injected with intraperitoneal SFN 15 minutes after the onset of ischaemia. SFN was found to increase brain HO-1 mRNA. The overall infarct volume was reduced in the treated group by 30% [152].

As discussed, microvascular spasm may contribute more to poor outcome after SAH than macroscopic spasm of the main arteries. Microvascular spasm is more difficult to study experimentally, and the effect of SFN on this has not been reported, though unlikely to respond differently to SFN, compared to macrovascular spasm. Whichever predominates, both occur in a delayed manner three days to three weeks after SAH, which offers a good therapeutic window for treatment unlike most other types of ischaemic stroke.

There are no published clinical studies of SFN in humans after SAH. Indeed, there are no studies of direct SFN administration in humans at all, due to its relatively short half-life making clinical administration problematic. It has therefore been studied in the context of cruciferous vegetables. Cruciferous vegetables of the genus Brassica, including broccoli, cauliflower, Brussels sprouts, kale, collards, kohlrabi, and mustard, are a rich source of precursors of isothiocyanates called glucosinolates [153]. After ingestion of these vegetables, glucosinolates are hydrolysed by myrosinase; these two components, normally stored in separate subcellular locations, are brought together during digestion [154, 155]. Also, microorganisms in the colon have been shown to be involved in hydrolysis of glucosinolates into isothiocyanates [156]. Amongst all the cruciferous vegetables, broccoli in particular contains significant amounts of 4-methylsulfinylbutyl glucosinolate (4-MSB) or glucoraphanin [157] which can subsequently be converted to SFN.

The bioavailability of SFN has been studied in animals and humans. Due to its lipophilicity and molecular size, SFN is likely to passively diffuse through enterocytes. It is easily absorbed, conjugated to glutathione, and metabolised via the mercapturic acid pathway sequentially producing cysteinylglycine (SFN-CG), cysteine (SFN-Cys), and N-acetyl-cysteine (SFN-NAC) conjugates which are excreted in the urine [158]. SFN is primarily absorbed in the jejunum, and its bioavailability in humans is 74% [159]. In both humans and rats, approximately 70% of orally administered SFN is eliminated via the mercapturic acid pathway within 12–24 hours [160].

SFN has been demonstrated in the gastrointestinal and genitourinary tracts as well as the liver, pancreas, lung, and heart, albeit in varying concentrations; bioactivity may differ amongst organs [161]. In order for SFN to exert its neuroprotective effect, good CNS penetration is vital. One mouse study showed that SFN crossed the BBB and was detectable in the cerebral tissue including the midbrain and striatum between 15 and 120 minutes after intraperitoneal injection [162]. Another study demonstrated that at two and six hours after oral gavage, SFN metabolites were detectable in the CNS. Concentrations were relatively low, and SFN itself was no longer detectable, but this may be due to the relatively late time points selected [163]. Pragmatically, all previous experimental SAH studies have demonstrated potential benefits with peripherally administered SFN, and although peak concentration may be brief, there is evidence that repetitive stimulation with SFN can lead to elevated target mRNA for 24 hours and proteins for 48 hours [138, 144, 150]. Hence, with regular SFN dosing, a sustained upregulation of the protective Nrf2 transcriptome might be expected.

In many respects, SFN would appear to be an excellent candidate as a new therapeutic for patients after SAH. The lack of a practical formulation for clinical use has prevented trials to date. However, SFX-01 (Evgen Pharma) represents a novel solution to this by complexing SFN with *α*-cyclodextrin to produce a stable powder that can be used clinically. A randomised controlled trial of SFX-01 after SAH is underway (NCT02614742).

### 5.1. Curcumin

Curcumin has been tested in several SAH models. In an *in vitro* SAH model where cortical neurons were exposed to OxyHb, curcumin was shown to reduce oxidative stress, inflammatory cytokines including TNF-*α*, IL-1*β*, and IL-6, and neural apoptosis [164].

In a SAH perforation model, curcumin was administered at the time of injury and one, three, and 24 hours later. It reduced inflammatory cytokines and the rate of vasospasm and DCI. There was no associated improvement in neurological recovery using rotarod and open-field activity assessment up to day three, despite observing a maximum effect of haemorrhagic infarct volume at day six. Perhaps further behavioural testing should have been performed at a later stage to address this. Interestingly, only a single dose at the time of haemorrhage was found to be associated with reduction in cerebral infarction at day six as well as reduced MCA diameter three days after the haemorrhage. Other treatment time points were not associated with these observed benefits [165].

Similar improvements in the rate of vasospasm were seen in a recent study comparing the actions of nimodipine, nicorandil, and low and high-dose curcumin on cerebral vasospasm. This demonstrated that high-dose curcumin is associated with a lower rate of vasospasm compared to nimodipine and nicorandil [166]. Unfortunately, this study did not perform any behavioural testing. In addition, there is ambiguity regarding the exact timing of the treatment in relation to surgery. Although it is mentioned that only a single dose was given to all animals, further clarification regarding the timing of intervention as well as time points of the different analyses performed would be helpful before drawing any conclusions.

Animal behaviour was examined in a rat double-haemorrhage model following intraperitoneal injection of curcumin three hours after SAH induction and daily thereafter for six days. Curcumin was shown to increase superoxide dismutase and catalase and reduce MDA levels in the cortex and hippocampus. Basilar artery perimeter and thickness were significantly altered in the treatment group indicating a reduction in vasospasm. There was reduced neuronal degeneration. Importantly, mortality was reduced and blinded neurological scores improved with curcumin. Curcumin-treated rats had a significantly lower mortality rate assessed during and after the induction of SAH compared to the other groups. Neurological scoring was performed at six hours and days one, three, five, and seven after the haemorrhage induction. Curcumin rats displayed better neurological scores up to day seven, but even in the untreated group, the neurological scores showed a positive trend on day seven [167]. This may suggest that the observed benefit may be lost if animals were to be assessed at a later time point. On the other hand, this finding could also represent the natural recovery from the disease.

Curcumin has been associated with an improvement in learning and memory impairment measured by the Morris water maze in a rat SAH model. Treatment duration with curcumin lasted for four weeks, and the authors claim that the positive benefit is secondary to downregulation of hippocampal TNF-*α* and inducible NOS. However, we were unable to make a full assessment of the manuscript as it is published in Chinese [168].

Curcumin has also been found to have benefits in ischaemic stroke similar to SFN. Nrf2 and HO-1 gene and protein levels were measured at three, six, 12, 24, 48, and 72 hours after MCA occlusion. An increase was seen at three hours, peaking at 24 hours poststroke. Infarct volume, brain water content, and early behavioural deficits assessed at 24 hours were reduced in the curcumin group [132].

### 5.2. Astaxanthin

Astaxantin (ASTX) is a carotenoid found in algae, fungi, complex plants, and seafood. It has been shown to be a powerful antioxidant [169]. The underlying mechanism of upregulation of Nrf2 by ASTX is not fully understood. However, it is thought that ASTX activates kinases such as phosphoinositol-3 kinase and extracellular signal-regulated protein kinase which in turn upregulates the Nrf2 pathway [170]. In an experimental SAH model, ASTX was administered intrathecally 30 minutes after the induction of SAH. Animals were sacrificed at 24 hours, and tissues were evaluated. ASTX was shown to upregulate the expression of enzymes regulated by Nrf2 including HO-1, NQO1, and GST-*α*1. Oxidative stress as measured by MDA levels was significantly reduced together with brain oedema, BBB disruption, and apoptosis. Neurological and behavioural deficits at 24 hours following SAH were improved [171]. These results were similar to their previous study which demonstrated the neuroprotective benefits of delayed treatment with oral ASTX, which started three hours post-SAH [172].

### 5.3. Lycopene

Lycopene is a natural carotenoid found mainly in tomatoes. It has multiple pleiotropic effects including antioxidant and anti-inflammatory actions [173] and neuroprotection from ischaemia [174]. At least some of its actions have been demonstrated to be due to upregulation of the Nrf2 pathway leading to neuroprotection in an experimental ischaemic model [175].

Lycopene has been tested in a rat SAH model. It was given once, two hours after SAH. Brain oedema, BBB disruption, and cortical apoptosis were significantly reduced at 24 hours. Neurology was only assessed at 24 hours, when neurological dysfunction was markedly reduced. The study showed a beneficial effect of lycopene due to reduction in inflammation as shown by downregulation of IL-1*β* and ICAM-1, but whether this was mediated through Nrf2 was not specifically investigated [176].

A phase II clinical trial assessing the effect of lycopene on cerebral vasospasm and autoregulation after SAH has been registered (NCT00905931). It has recruited 15 patients to date but is currently on hold due to temporary problems with IMP availability (personal communication).

### 5.4. Tetra-butyl Hydroquinone

Tetra-butyl hydroquinone (tBHQ) has been evaluated in two SAH models [177, 178]. In mice 24 hours after haemorrhage, there was no evidence of Nrf2 upregulation by tBHQ. However, at 48 hours after haemorrhage, tBHQ upregulated the expression of KEAP1, Nrf2, HO-1, NQO1, and GST*α*1 [178]. These animals displayed less brain oedema, BBB impairment, cortical apoptosis, and neurodegeneration. The treatment was started at two hours and repeated at 12, 24, and 36 hours after SAH. The study included two groups. The first group was decapitated at 48 hours, and tissues were evaluated. This showed marked upregulation of Nrf2 in neurons and glial cells. In the second experiment, the rats were trained and evaluated in a Morris water maze demonstrating significant improvement in performance and learning deficits following tBHQ on days four and five. Further memory testing up to day 8 showed no significant difference following the use of tBHQ [177].

### 5.5. Dimethyl Fumarate

Dimethyl fumarate (DMF) is an ester of fumaric acid conventionally used in the treatment of psoriasis [179]. DMF has been shown to modulate inflammation in the brain and specifically in multiple sclerosis (MS) through activation of the KEAP1-Nrf2-ARE pathway [180, 181]. Following a randomised phase III clinical trial showing that DMF ameliorated relapsing-remitting MS [182], it has been repurposed and is being used clinically for this indication.

The effects of DMF have been investigated in a rat prechiasmatic cistern injection model utilising autologous blood in rats. DMF was administered orally twice daily for two days, but the exact timing of administration relative to SAH was not specified. Two sets of experiments were performed. In the first group, tissue analysis took place only once 48 hours after surgery. The second experiment involved Morris water maze assessment of trained animals up to five days after the haemorrhage. Activities of KEAP1, Nrf2, and HO-1 were significantly increased within glial cells and neurons of animals treated with DMF. Cortical MDA was decreased, and superoxide dismutase and glutathione peroxidase activities were increased. Levels of proinflammatory cytokines IL-1*β*, TNF-*α*, and IL-6 were reduced. Behavioural assessments in the group treated with DMF showed marked improvement in the performance of the treatment group which was more evident on days four and five [183].

### 5.6. Melatonin

Melatonin is a well-known antioxidant with the ability to scavenge free radicals probably acting through multiple mechanisms [184, 185]. Studies have shown attenuation of early brain injury and vasospasm [186] with improvement in early neurological function (assessed at 48 hours) with a once daily regimen [187] and reduction in mortality rate (assessed within 24 hours of SAH induction) [188]. Furthermore, the mechanism behind these actions was linked to activation of Nrf2 [189].

In a rat SAH model, animals were treated with intraperitoneal injection of melatonin 150 mg/kg at two and 24 hours after the induction of SAH. Neurological scores and brain tissues were examined at 48 hours. Nrf2 and HO-1 were upregulated at 48 hours in the SAH group, mainly expressed on neurons. The levels of HO-1, NQO1, and GST-*α*1 mRNA were significantly increased in the cortex following treatment with melatonin. Brain oedema, BBB dysfunction, and cortical apoptosis were all reduced. Within the time frame of the experiment, early assessments of behavioural deficits of animals were significantly reduced in the treatment arm [189].

### 5.7. Erythropoietin

Erythropoietin (EPO) is a pleiotropic molecule with known effects on Nrf2. In an experimental SAH model, EPO was injected intraperitoneally five minutes after SAH and every eight hours up to 48 hours. HO-1, NQO1, and GST-*α*1 were all upregulated. Cortical apoptosis, brain oedema, and BBB impairment were all significantly reduced in the EPO-treated group. Although EPO is a pleiotropic molecule, the upregulation of Nrf2 proteins supports the mechanism of action through activation of the Nrf2-ARE pathway, hence reducing oxidative stress [190]. Other than its effects on early brain injury, further experimental studies in SAH made strong suggestions of potential benefits of EPO including improved cerebral blood flow [191, 192] autoregulation [192], reduction in vasoconstriction [193], post-SAH cerebral ischaemia [193, 194], and early improvement in behavioural function [193, 195, 196], and one study even claimed a reduction in mortality rate although this was only measured up to 72 hours [196].

EPO is unique amongst the agents identified in having been assessed in human studies. In a small case series of seven patients, EPO was shown to be effective in improving brain tissue oxygen tension if given over three consecutive days. This showed anti-inflammatory properties as well as restoration of cerebral autoregulation [197]. So far, two double-blinded placebo-controlled randomised trials [198, 199] have failed to demonstrate benefit with high-dose intravenous EPO. However, these studies were small (73 and 80 patients) and not adequately powered to show a clinical difference. Tseng et al. did observe a trend towards a reduced incidence of severe vasospasm, and a review concluded that EPO possibly reduces the severity of the cerebral vasospasm but not its incidence [200]. These trials have however demonstrated the safety of EPO following SAH and allayed any safety concerns over EPO and its association with increased risk of thrombosis [201]. The latter is important since SAH is a condition where patients are already in a hypercoagulable state and historically haemodilution has been advocated. However, further larger clinical trials would be required to address the efficacy of EPO.

### 5.8. Summary

There is good experimental evidence suggesting that early Nrf2 activation reduces deficits early after SAH although more studies examining their effects on long-term outcome are needed. The reasons underlying the paucity of studies examining long-term functional outcome are unclear. This may be due to poor experimental design, practical reasons, or difficulty in inducing significant late deficits without excessive early mortality in rodent SAH models. Other than EPO, there have been no completed human clinical trials of Nrf2 activation in SAH. Experimental studies suggest biochemical and early functional improvements following treatment, although it is difficult to test for the more subtle neurocognitive deficits most prevalent in patients with SAH. The timing of administration of first dose in animal studies was generally early (often within 2 hours of SAH), with few studies providing data on later use. Although this is a potential concern for human studies, even if data on later administration was available in animal models, extrapolation of the therapeutic window from animals to humans is notoriously difficult if not impossible, and given the generally much slower evolution of SAH in humans compared to rodents, trials administering at the earliest available opportunity, up to 72 hours after ictus when patients start to deteriorate, could be considered. There are a number of potential agents that could be used in this context. There are no head-to-head comparisons in the literature, and they are all reported to penetrate the CNS, have relatively good safety profiles, and with exception of EPO, can be given orally. There is therefore little to guide which may be most suitable.

## 6. Conclusion

Outcomes following SAH remain poor despite advances in treatment. The mechanisms underlying recovery from SAH are multifactorial; however, Nrf2 activation appears to play a key protective role. There is overwhelming evidence for the therapeutic potential of several Nrf2 activators, with studies replicated in different SAH models and different laboratories. In the absence of any human data, there is a clear need for clinical studies to examine the safety and efficacy of Nrf2 activation after SAH.

## Figures and Tables

**Table 1:** A summary of findings from experimental subarachnoid haemorrhage studies testing agents that activate the Nrf2 pathway, with relevant human data for these agents.

Agent	Curcumin	Astaxanthin	Lycopene	*tert*-Butyl hydroquinone	Dimethyl fumarate	Melatonin	Erythropoietin	Sulforaphane
Animal SAH model	Rat, mouse	Rat, rabbit	Rat	Rat	Rat	Rat	Rat, rabbit	Rat
Timing of administration	0-4 weeks	30 min-3 h	2 h	0-36 h	Twice daily for 2 d	0-48 h	0-72 h	30 min-72 h
Method of administration	IP	IT & oral	IP	IP & oral	Oral	IP	SC, IV, & IP	IP
Animal dose	150-600 mg/kg	0.01-75 mg/kg	40 mg/kg	12.5-50 mg/kg	15 mg/kg	15-150 mg/kg	400-1000 IU/kg	5 mg/kg
Time of tissue evaluation	Days 3-7	24-72 h	24 h	24-48 h	48 h	24-48 h	24-72 h	12-72 h
Time of clinical assessments	6 h—day 7	0-72 h	24 h	Day 0-8	Days 2-5	24-48 h	Days 0-16	72 h
Biochemical effect	Yes	Yes	Yes	Yes	Yes	Yes	Yes	Yes
Clinical effect	Yes	Yes	Yes	Yes	Yes	Yes	Yes	Yes
Reduced vasospasm	Yes	Yes	Not assessed	Not assessed	Not assessed	Yes	Yes	Yes
Method of administration in humans	Oral	Oral	Oral	Oral	Oral	Oral	IV	Oral
Half-life	6-7 h [202]	15.9 ± 5.3 h [203]	28-61 h [204]	20-24 h [205]	12 min [206]	1.8-2.1 h [207]	6-9 h [201]	2.4-2.6 h [208]
BBB permeability	Yes [209]	Yes [210]	Yes [211]	Yes [212]	Yes [213]	Yes [214]	Yes [215]	Yes [163]
Toxicity	None known [216]	None known [217]	None known [204]	None known [218]	Progressive multifocal leukoencephalopathy & painful dermatitis [219]	None known [220]	Polycythaemia & secondary stroke [201]	None known

Details of the experimental studies are shown in Table 2.

**Table 2:** Animal studies published in English, investigating Nrf2 activators in experimental SAH.

Agent	Study	Animal SAH model	Time of doses	Method of administration	Animal dose	Time of tissue evaluation	Time of clinical assessment	Biochemical effect	Clinical effect	Other effects	Vasospasm
Curcumin	Wakade et al. 2009 [165]	Mouse	0, 1, 3, & 24 h	IP	150/300 mg/kg	72 & 96 h	Days 0, 1, 2, & 3	Attenuation of COX-2, IL-1, IL-6, iNOS, TNF-*α*, ICAM-1, & VCAM-1; reduced lipid peroxidation; & superoxide production	No effect	Reduced cerebral infraction	Reduced vasospasm
	Kuo et al. 2011 [167]	Rat	3 h & then once daily for 6 days	IP	20 mg/kg	Day 7	6 h, days 1, 3, 5, & 7	Lower glutamate & MDA levels, preserved SOD, & catalase level	Reduced mortality & improved functional outcomes	None	Reduced vasospasm
	Aydin et al. 2017 [166]	Rat	Single dose	IP	150/300/600 mg/kg	Blood at 1 h, brain extraction unclear	None	Reduced IL-1, TNF-*α*, & IL-6	Not done	None	Reduced vasospasm

Astaxanthin	Zhang et al. 2014 [172]	Rat Rabbit	30 min IT, 3 h Oral	IT, PO	IT 0.01-0.1 mmol/l, PO 25/75 mg/kg	24 & 72 h	0, 24, 48, & 72 h	SOD & GSH levels reduced, MDA levels elevated	Neurological improvement only at 24 & 48 h	Reduced BBB permeability, cerebral oedema, & apoptosis and reduced caspase-3 expression	Not assessed
	Wu et al. 2014 [171]	Rat	30 min	IT	T 0.01-0.1 mmol/l	24 h	24 h	Increased expression of Nrf2, GST-*α*1, HO-1, & NQO-1, reduced MDA levels	Better performance at 24 h	Reduced BBB permeability, cerebral oedema, & apoptosis	Not assessed

Lycopene	Wu et al. 2015 [176]	Rat	2 h	IP	40 mg/kg	24 h	24 h	Downregulation of TNF-*α*, IL-1*β*, & ICAM-1	Improved neurological function	Lessened oedema, disruption of BBB, & cortical apoptosis	Not assessed

Tetra-butyl hydroquinone	Wang and Theeuwes 2014 [177]	Rat	2, 12, 24, & 36 h	PO	12.5 mg/kg	48 h	Days 0, 2, 3, 4, 5, 6, 7, & 8	Increased Keap1, Nrf2, & HO-1 expression; upregulation of GST-*α*1, HO-1, & NQO-1; reduced MDA levels; increased GSH-P & SOD levels	Improved performance & learning deficits on days 4 & 5	Reduced BBB permeability, cerebral oedema, & apoptosis	Not assessed
	Li et al. 2015 [178]	Mouse	0, 8, & 16 h	IP	50 mg/kg	24 h	24 h	Increased expression of Beclin-1 & the LC3-II to LC3-I ratio	Improvement in neurological deficits	BBB permeability, cerebral oedema, & neuronal degeneration were reduced	Not assessed

Dimethyl fumarate	Liu et al. 2015 [183]	Rat	Twice daily for 2 days	PO	15 mg/kg	48 h	Days 2, 3, 4, & 5	Decreased IL-1*β*, TNF-*α*, IL-6, SOD, MDA & GSH-P, HO-1, NQO1 & GST-*α*1 upregulated	Reduction of learning deficits	Brain oedema, cortical apoptosis & necrosis decreased	Not assessed

Melatonin	Aydin et al. 2005 [186]	Rabbit	0, 2, 12, 24, 36, & 48 h	IP	5 mg/kg	48 h	None	Reduced endothelial cellular apoptosis	Not assessed	Reduced cellular apoptosis	Reduced vasospasm
	Ayer et al. 2008 [188]	Rat	2 h	IP	15/150 mg/kg	24 h	24 h	No effect on MDA	Reduced mortality only	Cerebral oedema reduced	Not assessed
	Ersahin et al. 2009 [187]	Rat	0, 24, & 48 h	IP	10 mg/kg	48 h	48 h	Myeloperoxidase activity decreased, chemiluminescence values decrease, MDA decreased, & GSH was preserved	Improved neurological score	Cerebral oedema & BBB permeability reduced	Reduced vasospasm

Erythropoietin	Alafaci et al. 2000 [194]	Rabbit	5 min, 8, 16, & 24 h	IP	1000 IU/kg	24 h	None	Increased CSF EPO levels	Not assessed	Decreased neuronal damage	Not assessed
	Buemi et al. 2000 [196]	Rabbit	0	IP	1000 IU/kg	72 h	24, 48, & 72 h	No significant increase in CSF EPO concentration	Reduced mortality rate	None	Not assessed
	Grasso et al. 2002 [193]	Rabbit	5 min	IP	1000 IU/kg	72 h	72 h	Increase in CSF EPO concentration	Improved neurological score	Reduced ischaemic neuronal damage	Reduced vasospasm
	Springborg et al. 2002 [192]	Rat	0	SC	400 IU/kg	48 h	None	No biochemical effect assessed	Not assessed	Normalised autoregulation of cerebral blood flow	Not assessed
	Grasso et al. 2002 [195]	Rabbit	5 min, 8, 16, 24, 32, 40, 48, 56, 64, & 72 h	IP	1000 IU/kg	72 h	72 h	Lower S-100 protein concentration in CSF	Improved neurological function	Reduced neuronal damage	Not assessed
	Murphy et al. 2008 [191]	Rabbit	Days 0, 2, 4, & 6	IV	500/1500 IU/kg	24 h	Days 0, 2, 4, 7, 9, & 16	Increased haematocrit values	Reduced mortality rate	Improved cerebra blood flow, reduced cellular apoptosis	No change
	Zhang et al. 2010 [190]	Rat	15 min, 7, 16, 24, 32, 40, & 48 h	IP	1000 IU/kg	48 h	Not assessed	Increased Nrf2 & HO-1 expression, and upregulation of GST-*α*1, HO-1, & NQO-1	Not assessed	Reduced impairment of cerebral oedema, cortical apoptosis, & BBB permeability	Not assessed

Sulforaphane	Chen et al. 2011 [138]	Rat	30 min and 12 & 36 h	IP	5 mg/kg	12, 24, & 48 h	Not assessed	Increased Nrf2 & HO-1 expression and upregulation of GST-*α*1, HO-1, & NQO-1	Improved function at 48 h	Decreased cerebral oedema, BBB permeability, & cortical apoptosis	Not assessed
	Zhao et al. 2016 [144]	Rat	30 min and 24, 48, & 72 h	IP	5 mg/kg	72 h	72 h	Increased Nrf2 & HO-1 expression; upregulation of GST-*α*1, HO-1, & NQO1; and decreased IL-1*β*, TNF-*α*, & IL-6	Reduced behavioural deficits	None	Reduced vasospasm

## Abbreviations

ARE: Antioxidant response element
BACH1: BTB and CNC homology 1
CBP: CREB-binding protein
HO-1: Heme-oxygenase 1
HP: Haptoglobin
KEAP1: Kelch-like ECH-associated protein 1
MAF: Musculoaponeurotic fibrosarcoma
Nrf2: Nuclear factor-erythroid 2- (NF-E2-) related factor 2
P: Phosphate group
PG: Prostaglandin
S(R): Sulphide side chain reduced by a group R
SH: Sulfhydryl side chain.
